# Supplementary material for: Multi-bit quantum random number generation from a single qubit quantum walk
Source: Sci Rep. 2019 Aug 23;9:12323. doi: 10.1038/s41598-019-48844-4 (PMC6707389; doi:10.1038/s41598-019-48844-4)
Supplement: Supplementary file 1 — Appendix [file 41598_2019_48844_MOESM1_ESM.pdf]

Appendix : Multi-bit quantum random number generation from a single qubit quantum walk

Anupam Sarkar[1,2] and C. M. Chandrashekar[1,2,*]
[1]The Institute of Mathematical Sciences, C. I. T. Campus, Taramani, Chennai 600113, India
[2]Homi Bhabha National Institute, Training School Complex, Anushakti Nagar, Mumbai 400094, India

Appendix A: Calculating Randomness of The State After First Step

1. Coin space

The initial state of the walker, $|\psi_{in}\rangle = (\cos\delta\,|\uparrow\rangle + e^{i\eta}\sin\delta\,|\downarrow\rangle)\otimes|0\rangle$. The Coin operation we consider

$$C(\theta) = \begin{bmatrix} \cos\theta & -i\sin\theta \\ -i\sin\theta & \cos\theta \end{bmatrix}$$

Therefore,

$$\Big[C(\theta)\otimes\mathbb{1}\Big]\,|\psi_{in}\rangle = (\cos\theta\cos\delta - ie^{i\eta}\sin\theta\sin\delta)\,|\uparrow\rangle + (e^{i\eta}\cos\theta\sin\delta - i\sin\theta\cos\delta)\,|\downarrow\rangle$$
$$S\Big[C(\theta)\otimes\mathbb{1}\Big]\,|\psi_{in}\rangle = (\cos\theta\cos\delta - ie^{i\eta}\sin\theta\sin\delta)\,|\uparrow\rangle\otimes|-1\rangle + (e^{i\eta}\cos\theta\sin\delta - i\sin\theta\cos\delta)\,|\downarrow\rangle\otimes|1\rangle$$

After one step the state of the walker,

$$|\psi_1\rangle = S\Big[C(\theta)\otimes\mathbb{1}\Big]\,|\psi_{in}\rangle = (\cos\theta\cos\delta - ie^{i\eta}\sin\theta\sin\delta)\,|\uparrow\rangle\otimes|-1\rangle + (e^{i\eta}\cos\theta\sin\delta - i\sin\theta\cos\delta)\,|\downarrow\rangle\otimes|1\rangle$$

So the corresponding density matrix ρ_1 will be,

$$\begin{aligned}
\rho_1 &= |\psi_1\rangle\langle\psi_1| \\
&= \Big[(\cos\theta\cos\delta - ie^{i\eta}\sin\theta\sin\delta)\,|\uparrow\rangle\otimes|-1\rangle + (e^{i\eta}\cos\theta\sin\delta - i\sin\theta\cos\delta)\,|\downarrow\rangle\otimes|1\rangle\Big]\times \\
&\Big[(\cos\theta\cos\delta + ie^{-i\eta}\sin\theta\sin\delta)\,\langle\uparrow|\otimes\langle-1| + (e^{-i\eta}\cos\theta\sin\delta + i\sin\theta\cos\delta)\,\langle\downarrow|\otimes\langle 1|\Big] \\
&= \Big(\cos^2\theta\cos^2\; - ie^{i\eta}\sin\theta\cos\theta\cos\delta\sin\delta + ie^{-i\eta}\sin\theta\cos\theta\sin\delta\cos\delta + \sin^2\theta\sin^2\delta\Big)\,|\uparrow\rangle\langle\uparrow|\otimes|-1\rangle\langle-1| \\
&+ \Big(e^{-i\eta}\cos^2\theta\sin\delta\cos\delta + i\sin\theta\cos\theta\cos^2\delta - i\sin\theta\cos\theta\sin^2\delta + e^{i\eta}\sin^2\theta\sin\delta\cos\delta\Big)\,|\uparrow\rangle\langle\downarrow|\otimes|-1\rangle\langle 1| \\
&+ \Big(e^{i\eta}\cos^2\theta\sin\delta\cos\delta + i\sin\theta\cos\theta\sin^2\delta - i\sin\theta\cos\theta\cos^2\delta + e^{-i\eta}\sin^2\theta\sin\delta\cos\delta\Big)\,|\downarrow\rangle\langle\uparrow|\otimes|1\rangle\langle-1| \\
&+ \Big(\cos^2\theta\sin^2\delta + ie^{i\eta}\sin\theta\cos\theta\sin\delta\cos\delta - ie^{-i\eta}\sin\theta\cos\theta\sin\delta\cos\delta + \sin^2\theta\cos^2\delta\Big)\,|\downarrow\rangle\langle\downarrow|\otimes|1\rangle\langle 1|
\end{aligned}$$

Now by tracing out the position space we'll get the reduced density matrix of the coin space, denoted by ρ_1^c

$$\begin{aligned}
\rho_1^c &= Tr_p(\rho_1) \\
&= \Big(\cos^2\theta\cos^2\delta - ie^{i\eta}\sin\theta\cos\theta\cos\delta\sin\delta + ie^{-i\eta}\sin\theta\cos\theta\sin\delta\cos\delta + \sin^2\theta\sin^2\delta\Big)\,|\uparrow\rangle\langle\uparrow| \\
&+ \Big(\cos^2\theta\sin^2\delta + ie^{i\eta}\sin\theta\cos\theta\sin\delta\cos\delta - ie^{-i\eta}\sin\theta\cos\theta\sin\delta\cos\delta + \sin^2\theta\cos^2\delta\Big)\,|\downarrow\rangle\langle\downarrow|
\end{aligned}$$

By simplifying the expression above we get the probability of obtaining $|\uparrow\rangle$ coin state, following the same pocedure to find the randomness in initial sate

$$P_\uparrow = \Big[(\cos\theta\cos\delta + \sin\theta\sin\delta)^2 - 2\sin\theta\cos\theta\sin\delta\cos\delta(1-\sin\eta)\Big]$$

Similarly for the coin state $|\downarrow\rangle$,

$$P_\downarrow = \Big[(\cos\theta\sin\delta + \sin\theta\cos\delta)^2 - 2\sin\theta\cos\theta\sin\delta\cos\delta(1+\sin\eta)\Big]$$

So the amount of randomness inherited by the state after 1 step quantum walk, denoted by R_i,

$$\begin{aligned}
R_i(\rho_1^c) = &- \left(P_\uparrow \ln P_\uparrow + P_\downarrow \ln P_\downarrow\right)\\
= &- \Bigg[\Big[(\cos\theta\cos\delta + \sin\theta\sin\delta)^2 - 2\sin\theta\cos\theta\sin\delta\cos\delta(1-\sin\eta)\Big]\\
&\times \ln\Big[(\cos\theta\cos\delta + \sin\theta\sin\delta)^2 - 2\sin\theta\cos\theta\sin\delta\cos\delta(1-\sin\eta)\Big]\\
&+ \Big[(\cos\theta\sin\delta + \sin\theta\cos\delta)^2 - 2\sin\theta\cos\theta\sin\delta\cos\delta(1+\sin\eta)\Big]\\
&\times \ln\Big[(\cos\theta\sin\delta + \sin\theta\cos\delta)^2 - 2\sin\theta\cos\theta\sin\delta\cos\delta(1+\sin\eta)\Big]\Bigg]
\end{aligned}$$

2. Position space

The density matrix after first step

$$\begin{aligned}
&\rho_1 = |\psi_1\rangle\langle\psi_1|\\
&= \Big(\cos^2\theta\cos^2 - ie^{i\eta}\sin\theta\cos\theta\cos\delta\sin\delta + ie^{-i\eta}\sin\theta\cos\theta\sin\delta\cos\delta + \sin^2\theta\sin^2\delta\Big)|\uparrow\rangle\langle\uparrow| \otimes |-1\rangle\langle -1|\\
&+ \Big(e^{-i\eta}\cos^2\theta\sin\delta\cos\delta + i\sin\theta\cos\theta\cos^2\delta - i\sin\theta\cos\theta\sin^2\delta + e^{i\eta}\sin^2\theta\sin\delta\cos\delta\Big)|\uparrow\rangle\langle\downarrow| \otimes |-1\rangle\langle 1|\\
&+ \Big(e^{i\eta}\cos^2\theta\sin\delta\cos\delta + i\sin\theta\cos\theta\sin^2\delta - i\sin\theta\cos\theta\cos^2\delta + e^{-i\eta}\sin^2\theta\sin\delta\cos\delta\Big)|\downarrow\rangle\langle\uparrow| \otimes |1\rangle\langle -1|\\
&+ \Big(\cos^2\theta\sin^2\delta + ie^{i\eta}\sin\theta\cos\theta\sin\delta\cos\delta - ie^{-i\eta}\sin\theta\cos\theta\sin\delta\cos\delta + \sin^2\theta\cos^2\delta\Big)|\downarrow\rangle\langle\downarrow| \otimes |1\rangle\langle 1|
\end{aligned}$$

Tracing out the coin space resulting position space density matrix would be

$$\begin{aligned}
&\rho_1^p = Tr_c(\rho_1)\\
&= \Big(\cos^2\theta\cos^2\delta - ie^{i\eta}\sin\theta\cos\theta\cos\delta\sin\delta + ie^{-i\eta}\sin\theta\cos\theta\sin\delta\cos\delta + \sin^2\theta\sin^2\delta\Big)|-1\rangle\langle -1|\\
&+ \Big(\cos^2\theta\sin^2\delta + ie^{i\eta}\sin\theta\cos\theta\sin\delta\cos\delta - ie^{-i\eta}\sin\theta\cos\theta\sin\delta\cos\delta + \sin^2\theta\cos^2\delta\Big)|1\rangle\langle 1|
\end{aligned}$$

Therefore the amount of randomness contained in the state,

$$\begin{aligned}
R_i(\rho_1^p) = &- \Bigg[\Big[(\cos\theta\cos\delta + \sin\theta\sin\delta)^2 - 2\sin\theta\cos\theta\sin\delta\cos\delta(1-\sin\eta)\Big]\\
&\times \ln\Big[(\cos\theta\cos\delta + \sin\theta\sin\delta)^2 - 2\sin\theta\cos\theta\sin\delta\cos\delta(1-\sin\eta)\Big]\\
&+ \Big[(\cos\theta\sin\delta + \sin\theta\cos\delta)^2 - 2\sin\theta\cos\theta\sin\delta\cos\delta(1+\sin\eta)\Big]\\
&\times \ln\Big[(\cos\theta\sin\delta + \sin\theta\cos\delta)^2 - 2\sin\theta\cos\theta\sin\delta\cos\delta(1+\sin\eta)\Big]\Bigg]
\end{aligned}$$

Interesting thing is to notice that after first step, amount of quantified randomness in position space and coin space are the same which is physically reasonable because of the fact that degees of freedom for coin and position space is same i.e two. Therefore the corresponding probability amplitudes for the two degrees of freedom contribute in the randomness as the result randomness corresponding two spaces will be same.

Appendix B: Calculating Randomness of The State After Second Step

1. Coin space

At the start of the second step the state of the walker is,

$$|\psi_1\rangle = (\cos\theta\cos\delta - ie^{i\eta}\sin\theta\sin\delta)\left|\uparrow\right\rangle\otimes|-1\rangle + (e^{i\eta}\cos\theta\sin\delta - i\sin\theta\cos\delta)\left|\downarrow\right\rangle\otimes|1\rangle$$

$$\Big[C(\theta)\otimes\mathbb{1}\Big]|\psi_1\rangle = \Big[(\cos^2\theta\cos\delta - ie^{i\eta}\sin\theta\cos\theta\sin\delta)\left|\uparrow\right\rangle\otimes|-1\rangle + (-i\sin\theta\cos\theta\cos\delta - e^{i\eta}\sin^2\theta\sin\delta)\left|\downarrow\right\rangle\otimes|-1\rangle$$
$$+ (-ie^{i\eta}\sin\theta\cos\theta\sin\delta - \sin^2\theta\cos\delta)\left|\uparrow\right\rangle\otimes|1\rangle + (e^{i\eta}\cos^2\theta\sin\delta - i\sin\theta\cos\theta\cos\delta)\left|\downarrow\right\rangle\otimes|1\rangle\Big]$$

$$S\Big[C(\theta)\otimes\mathbb{1}\Big]|\psi_1\rangle = |\psi_2\rangle = \Big[(\cos^2\theta\cos\delta - ie^{i\eta}\sin\theta\cos\theta\sin\delta)\left|\uparrow\right\rangle\otimes|-2\rangle + (-i\sin\theta\cos\theta\cos\delta - e^{i\eta}\sin^2\theta\sin\delta)\left|\downarrow\right\rangle\otimes|0\rangle$$
$$+ (-ie^{i\eta}\sin\theta\cos\theta\sin\delta - \sin^2\theta\cos\delta)\left|\uparrow\right\rangle\otimes|0\rangle + (e^{i\eta}\cos^2\theta\sin\delta - i\sin\theta\cos\theta\cos\delta)\left|\downarrow\right\rangle\otimes|2\rangle\Big]$$

The density matrix denoted by ρ_2

$$\rho_2 = |\psi_2\rangle\langle\psi_2| = \Big[(\cos^2\theta\cos\delta - ie^{i\eta}\sin\theta\cos\theta\sin\delta)\left|\uparrow\right\rangle\otimes|-2\rangle + (-i\sin\theta\cos\theta\cos\delta - e^{i\eta}\sin^2\theta\sin\delta)\left|\downarrow\right\rangle\otimes|0\rangle$$
$$+ (-ie^{i\eta}\sin\theta\cos\theta\sin\delta - \sin^2\theta\cos\delta)\left|\uparrow\right\rangle\otimes|0\rangle + (e^{i\eta}\cos^2\theta\sin\delta - i\sin\theta\cos\theta\cos\delta)\left|\downarrow\right\rangle\otimes|2\rangle\Big]$$
$$\Big[(\cos^2\theta\cos\delta + ie^{-i\eta}\sin\theta\cos\theta\sin\delta)\left\langle\uparrow\right|\otimes\langle-2| + (i\sin\theta\cos\theta\cos\delta - e^{-i\eta}\sin^2\theta\sin\delta)\left\langle\downarrow\right|\otimes\langle 0|$$
$$+ (ie^{-i\eta}\sin\theta\cos\theta\sin\delta - \sin^2\theta\cos\delta)\left\langle\uparrow\right|\otimes\langle 0| + (e^{-i\eta}\cos^2\theta\sin\delta + i\sin\theta\cos\theta\cos\delta)\left\langle\downarrow\right|\otimes\langle 2|\Big]$$

$$= \Big[(\cos^4\theta\cos^2\delta + ie^{-i\eta}\sin\theta\cos^3\theta\sin\delta\cos\delta - ie^{i\eta}\sin\theta\cos^3\theta\sin\delta\cos\delta + \sin^2\theta\cos^2\theta\sin^2\delta)\left|\uparrow\right\rangle\left\langle\uparrow\right|\otimes|-2\rangle\langle-2|$$
$$+ (i\sin\theta\cos^3\theta\cos^2\delta - e^{-i\eta}\sin^2\theta\cos^2\theta\sin\delta\cos\delta + e^{i\eta}\sin^2\theta\cos^2\theta\sin\delta\cos\delta + i\sin^3\theta\cos\theta\sin^2\delta)\left|\uparrow\right\rangle\left\langle\downarrow\right|\otimes|-2\rangle\langle 0|$$
$$+ (ie^{-i\eta}\sin\theta\cos^3\theta\cos\delta\sin\delta - \sin^2\theta\cos^2\theta\cos^2\delta + \sin^2\theta\cos^2\theta\sin^2\delta + ie^{i\eta}\sin^3\theta\cos\theta\sin\delta\cos\delta)\left|\uparrow\right\rangle\left\langle\uparrow\right|\otimes|-2\rangle\langle 0|$$
$$+ (e^{-i\eta}\cos^4\theta\cos\delta\sin\delta + i\sin\theta\cos^3\theta\cos^2\delta - i\sin\theta\cos^3\theta\sin^2\delta + e^{i\eta}\sin^2\theta\cos^2\theta\sin\delta\cos\delta)\left|\uparrow\right\rangle\left\langle\downarrow\right|\otimes|-2\rangle\langle 2|$$
$$+ (-i\sin\theta\cos^3\theta\cos^2\delta + e^{-i\eta}\sin^2\theta\cos^2\theta\sin\delta\cos\delta - e^{i\eta}\sin^2\theta\cos^2\theta\sin\delta\cos\delta - i\sin^3\theta\cos\theta\sin^2\delta)\left|\downarrow\right\rangle\left\langle\uparrow\right|\otimes|0\rangle\langle-2|$$
$$+ (\sin^2\theta\cos^2\theta\cos^2\delta + ie^{-i\eta}\sin^3\theta\cos\theta\sin\delta\cos\delta - ie^{i\eta}\sin^3\theta\cos\theta\sin\delta\cos\delta + \sin^4\theta\sin^2\delta)\left|\downarrow\right\rangle\left\langle\downarrow\right|\otimes|0\rangle\langle 0|$$
$$+ (e^{-i\eta}\sin^2\theta\cos^2\theta\sin\delta\cos\delta - i\sin^3\theta\cos\theta\cos^2\delta - i\sin^3\theta\cos\theta\sin^2\delta + e^{i\eta}\sin^4\theta\sin\delta\cos\delta)\left|\downarrow\right\rangle\left\langle\uparrow\right|\otimes|0\rangle\langle 0|$$
$$+ (-ie^{-i\eta}\sin\theta\cos^3\theta\sin\delta\cos\delta + \sin^2\theta\cos^2\theta\cos^2\delta - \sin^2\theta\cos^2\theta\sin^2\delta - ie^{i\eta}\sin^3\theta\cos\theta\sin\delta\cos\delta)\left|\downarrow\right\rangle\left\langle\downarrow\right|\otimes|0\rangle\langle 2|$$
$$+ (-ie^{i\eta}\sin\theta\cos^3\theta\sin\delta\cos\delta + \sin^2\theta\cos^2\theta\sin^2\delta - \sin^2\theta\cos^2\theta\cos^2\delta - ie^{-i\eta}\sin^3\theta\cos\theta\sin\delta\cos\delta)\left|\uparrow\right\rangle\left\langle\uparrow\right|\otimes|0\rangle\langle-2|$$
$$+ (e^{i\eta}\sin^2\theta\cos^2\theta\sin\delta\cos\delta + i\sin^3\theta\cos\theta\sin^2\delta - i\sin^3\theta\cos\theta\cos^2\delta + e^{-i\eta}\sin^4\theta\sin\delta\cos\delta)\left|\uparrow\right\rangle\left\langle\downarrow\right|\otimes|0\rangle\langle 0|$$
$$+ (\sin^2\theta\cos^2\theta\sin^2\delta + ie^{i\eta}\sin^3\theta\cos\theta\sin\delta\cos\delta - ie^{-i\eta}\sin^3\theta\cos\theta\sin\delta\cos\delta + \sin^4\theta\cos^2\delta)\left|\uparrow\right\rangle\left\langle\uparrow\right|\otimes|0\rangle\langle 0|$$
$$+ (-i\sin\theta\cos^3\theta\sin^2\delta + e^{i\eta}\sin^2\theta\cos^2\theta\sin\delta\cos\delta - e^{-i\eta}\sin^2\theta\cos^2\theta\sin\delta\cos\delta - i\sin^3\theta\cos\theta\cos^2\delta)\left|\uparrow\right\rangle\left\langle\downarrow\right|\otimes|0\rangle\langle 2|$$
$$+ (e^{i\eta}\cos^4\theta\sin\delta\cos\delta + i\sin\theta\cos^3\theta\sin^2\delta - i\sin\theta\cos^3\theta\cos^2\delta + e^{-i\eta}\sin^2\theta\cos^2\theta\sin\delta\cos\delta)\left|\downarrow\right\rangle\left\langle\uparrow\right|\otimes|2\rangle\langle-2|$$
$$+ (ie^{i\eta}\sin\theta\cos^3\theta\sin\delta\cos\delta - \sin^2\theta\cos^2\theta\sin^2\delta + \sin^2\theta\cos^2\theta\cos^2\delta + ie^{-i\eta}\sin^3\theta\cos\theta\sin\delta\cos\delta)\left|\downarrow\right\rangle\left\langle\downarrow\right|\otimes|2\rangle\langle 0|$$
$$+ (i\sin\theta\cos^3\theta\sin^2\delta - e^{i\eta}\sin^2\theta\cos^2\theta\sin\delta\cos\delta + e^{-i\eta}\sin^2\theta\cos^2\theta\sin\delta\cos\delta + i\sin^3\theta\cos\theta\cos^2\delta)\left|\downarrow\right\rangle\left\langle\uparrow\right|\otimes|2\rangle\langle 0|$$
$$+ (\cos^4\theta\sin^2\delta + ie^{i\eta}\sin\theta\cos^3\theta\sin\delta\cos\delta - ie^{-i\eta}\sin\theta\cos^3\theta\sin\delta\cos\delta + \sin^2\theta\cos^2\theta\cos^2\delta)\left|\downarrow\right\rangle\left\langle\downarrow\right|\otimes|2\rangle\langle 2|\Big]$$

Now the reduced density matrix for the coin space

$$\rho_2^c = \Big[(\cos^4\theta\cos^2\delta + ie^{-i\eta}\sin\theta\cos^3\theta\sin\delta\cos\delta - ie^{i\eta}\sin\theta\cos^3\theta\sin\delta\cos\delta + \sin^2\theta\cos^2\theta\sin^2\delta)\left|\uparrow\right\rangle\left\langle\uparrow\right|$$
$$+ +(\sin^2\theta\cos^2\theta\cos^2\delta + ie^{-i\eta}\sin^3\theta\cos\theta\sin\delta\cos\delta - ie^{i\eta}\sin^3\theta\cos\theta\sin\delta\cos\delta + \sin^4\theta\sin^2\delta)\left|\downarrow\right\rangle\left\langle\downarrow\right|$$
$$+ (\sin^2\theta\cos^2\theta\sin^2\delta + ie^{i\eta}\sin^3\theta\cos\theta\sin\delta\cos\delta - ie^{-i\eta}\sin^3\theta\cos\theta\sin\delta\cos\delta + \sin^4\theta\cos^2\delta)\left|\uparrow\right\rangle\left\langle\uparrow\right|$$
$$+ (\cos^4\theta\sin^2\delta + ie^{i\eta}\sin\theta\cos^3\theta\sin\delta\cos\delta - ie^{-i\eta}\sin\theta\cos^3\theta\sin\delta\cos\delta + \sin^2\theta\cos^2\theta\cos^2\delta)\left|\downarrow\right\rangle\left\langle\downarrow\right|\Big]$$

By simplifying the above expression, we get the form

$$\begin{aligned}\rho_2^c =&(\cos^4\theta\cos^2\delta + \sin\eta\sin 2\theta\cos 2\theta\sin\delta\cos\delta + 2\sin^2\theta\cos^2\theta\sin^2\delta + \sin^4\theta\cos^2\delta)\left|\uparrow\right\rangle\left\langle\uparrow\right| \\ &+ (\cos^4\theta\sin^2\delta - \sin\eta\sin 2\theta\cos 2\theta\sin\delta\cos\delta + 2\sin^2\theta\cos^2\theta\cos^2\delta + \sin^4\theta\sin^2\delta)\left|\downarrow\right\rangle\left\langle\downarrow\right|\end{aligned}$$

Therefore,

$$\begin{aligned}R_i(\rho_2^c) = -\Bigg[&\Big[(\cos^4\theta\cos^2\delta + \sin\eta\sin 2\theta\cos 2\theta\sin\delta\cos\delta + 2\sin^2\theta\cos^2\theta\sin^2\delta + \sin^4\theta\cos^2\delta) \\ &\ln((\cos^4\theta\cos^2\delta + \sin\eta\sin 2\theta\cos 2\theta\sin\delta\cos\delta + 2\sin^2\theta\cos^2\theta\sin^2\delta + \sin^4\theta\cos^2\delta\Big] \\ &+\Big[(\cos^4\theta\sin^2\delta - \sin\eta\sin 2\theta\cos 2\theta\sin\delta\cos\delta + 2\sin^2\theta\cos^2\theta\cos^2\delta + \sin^4\theta\sin^2\delta) \\ &\ln((\cos^4\theta\sin^2\delta - \sin\eta\sin 2\theta\cos 2\theta\sin\delta\cos\delta + 2\sin^2\theta\cos^2\theta\cos^2\delta + \sin^4\theta\sin^2\delta))\Big]\Bigg]\end{aligned}$$

2. Position space

We'll use the expression of the density matrix after second step derived above.

$$\begin{aligned}\rho_2 =& \left|\psi_2\right\rangle\left\langle\psi_2\right| \\ =& \Big[(\cos^4\theta\cos^2\delta + ie^{-i\eta}\sin\theta\cos^3\theta\sin\delta\cos\delta - ie^{i\eta}\sin\theta\cos^3\theta\sin\delta\cos\delta + \sin^2\theta\cos^2\theta\sin^2\delta)\left|\uparrow\right\rangle\left\langle\uparrow\right|\otimes\left|-2\right\rangle\left\langle-2\right| \\ &+ (i\sin\theta\cos^3\theta\cos^2\delta - e^{-i\eta}\sin^2\theta\cos^2\theta\sin\delta\cos\delta + e^{i\eta}\sin^2\theta\cos^2\theta\sin\delta\cos\delta + i\sin^3\theta\cos\theta\sin^2\delta)\left|\uparrow\right\rangle\left\langle\downarrow\right|\otimes\left|-2\right\rangle\left\langle 0\right| \\ &+ (ie^{-i\eta}\sin\theta\cos^3\theta\cos\delta\sin\delta - \sin^2\theta\cos^2\theta\cos^2\delta + \sin^2\theta\cos^2\theta\sin^2\delta + ie^{i\eta}\sin^3\theta\cos\theta\sin\delta\cos\delta)\left|\uparrow\right\rangle\left\langle\uparrow\right|\otimes\left|-2\right\rangle\left\langle 0\right| \\ &+ (e^{-i\eta}\cos^4\theta\cos\delta\sin\delta + i\sin\theta\cos^3\theta\cos^2\delta - i\sin\theta\cos^3\theta\sin^2\delta + e^{i\eta}\sin^2\theta\cos^2\theta\sin\delta\cos\delta)\left|\uparrow\right\rangle\left\langle\downarrow\right|\otimes\left|-2\right\rangle\left\langle 2\right| \\ &+ (-i\sin\theta\cos^3\theta\cos^2\delta + e^{-i\eta}\sin^2\theta\cos^2\theta\sin\delta\cos\delta - e^{i\eta}\sin^2\theta\cos^2\theta\sin\delta\cos\delta - i\sin^3\theta\cos\theta\sin^2\delta)\left|\downarrow\right\rangle\left\langle\uparrow\right|\otimes\left|0\right\rangle\left\langle-2\right| \\ &+ (\sin^2\theta\cos^2\theta\cos^2\delta + ie^{-i\eta}\sin^3\theta\cos\theta\sin\delta\cos\delta - ie^{i\eta}\sin^3\theta\cos\theta\sin\delta\cos\delta + \sin^4\theta\sin^2\delta)\left|\downarrow\right\rangle\left\langle\downarrow\right|\otimes\left|0\right\rangle\left\langle 0\right| \\ &+ (e^{-i\eta}\sin^2\theta\cos^2\theta\sin\delta\cos\delta - i\sin^3\theta\cos\theta\cos^2\delta - i\sin^3\theta\cos\theta\sin^2\delta + e^{i\eta}\sin^4\theta\sin\delta\cos\delta)\left|\downarrow\right\rangle\left\langle\uparrow\right|\otimes\left|0\right\rangle\left\langle 0\right| \\ &+ (-ie^{-i\eta}\sin\theta\cos^3\theta\sin\delta\cos\delta + \sin^2\theta\cos^2\theta\cos^2\delta - \sin^2\theta\cos^2\theta\sin^2\delta - ie^{i\eta}\sin^3\theta\cos\theta\sin\delta\cos\delta)\left|\downarrow\right\rangle\left\langle\downarrow\right|\otimes\left|0\right\rangle\left\langle 2\right| \\ &+ (-ie^{i\eta}\sin\theta\cos^3\theta\sin\delta\cos\delta + \sin^2\theta\cos^2\theta\sin^2\delta - \sin^2\theta\cos^2\theta\cos^2\delta - ie^{-i\eta}\sin^3\theta\cos\theta\sin\delta\cos\delta)\left|\uparrow\right\rangle\left\langle\uparrow\right|\otimes\left|0\right\rangle\left\langle-2\right| \\ &+ (e^{i\eta}\sin^2\theta\cos^2\theta\sin\delta\cos\delta + i\sin^3\theta\cos\theta\sin^2\delta - i\sin^3\theta\cos\theta\cos^2\delta + e^{-i\eta}\sin^4\theta\sin\delta\cos\delta)\left|\uparrow\right\rangle\left\langle\downarrow\right|\otimes\left|0\right\rangle\left\langle 0\right| \\ &+ (\sin^2\theta\cos^2\theta\sin^2\delta + ie^{i\eta}\sin^3\theta\cos\theta\sin\delta\cos\delta - ie^{-i\eta}\sin^3\theta\cos\theta\sin\delta\cos\delta + \sin^4\theta\cos^2\delta)\left|\uparrow\right\rangle\left\langle\uparrow\right|\otimes\left|0\right\rangle\left\langle 0\right| \\ &+ (-i\sin\theta\cos^3\theta\sin^2\delta + e^{i\eta}\sin^2\theta\cos^2\theta\sin\delta\cos\delta - e^{-i\eta}\sin^2\theta\cos^2\theta\sin\delta\cos\delta - i\sin^3\theta\cos\theta\cos^2\delta)\left|\uparrow\right\rangle\left\langle\downarrow\right|\otimes\left|0\right\rangle\left\langle 2\right| \\ &+ (e^{i\eta}\cos^4\theta\sin\delta\cos\delta + i\sin\theta\cos^3\theta\sin^2\delta - i\sin\theta\cos^3\theta\cos^2\delta + e^{-i\eta}\sin^2\theta\cos^2\theta\sin\delta\cos\delta)\left|\downarrow\right\rangle\left\langle\uparrow\right|\otimes\left|2\right\rangle\left\langle-2\right| \\ &+ (ie^{i\eta}\sin\theta\cos^3\theta\sin\delta\cos\delta - \sin^2\theta\cos^2\theta\sin^2\delta + \sin^2\theta\cos^2\theta\cos^2\delta + ie^{-i\eta}\sin^3\theta\cos\theta\sin\delta\cos\delta)\left|\downarrow\right\rangle\left\langle\downarrow\right|\otimes\left|2\right\rangle\left\langle 0\right| \\ &+ (i\sin\theta\cos^3\theta\sin^2\delta - e^{i\eta}\sin^2\theta\cos^2\theta\sin\delta\cos\delta + e^{-i\eta}\sin^2\theta\cos^2\theta\sin\delta\cos\delta + i\sin^3\theta\cos\theta\cos^2\delta)\left|\downarrow\right\rangle\left\langle\uparrow\right|\otimes\left|2\right\rangle\left\langle 0\right| \\ &+ (\cos^4\theta\sin^2\delta + ie^{i\eta}\sin\theta\cos^3\theta\sin\delta\cos\delta - ie^{-i\eta}\sin\theta\cos^3\theta\sin\delta\cos\delta + \sin^2\theta\cos^2\theta\cos^2\delta)\left|\downarrow\right\rangle\left\langle\downarrow\right|\otimes\left|2\right\rangle\left\langle 2\right|\Big]\end{aligned}$$

Now reduced density matrix of position space will be,

$$\rho_2^p = \Big[(\cos^4\theta\cos^2\delta + ie^{-i\eta}\sin\theta\cos^3\theta\sin\delta\cos\delta - ie^{i\eta}\sin\theta\cos^3\theta\sin\delta\cos\delta + \sin^2\theta\cos^2\theta\sin^2\delta)\left|-2\right\rangle\left\langle -2\right|$$
$$+ (ie^{-i\eta}\sin\theta\cos^3\theta\cos\delta\sin\delta - \sin^2\theta\cos^2\theta\cos^2\delta + \sin^2\theta\cos^2\theta\sin^2\delta + ie^{i\eta}\sin^3\theta\cos\theta\sin\delta\cos\delta)\left|-2\right\rangle\left\langle 0\right|$$
$$+ (\sin^2\theta\cos^2\theta\cos^2\delta + ie^{-i\eta}\sin^3\theta\cos\theta\sin\delta\cos\delta - ie^{i\eta}\sin^3\theta\cos\theta\sin\delta\cos\delta + \sin^4\theta\sin^2\delta)\left|0\right\rangle\left\langle 0\right|$$
$$+ (-ie^{-i\eta}\sin\theta\cos^3\theta\sin\delta\cos\delta + \sin^2\theta\cos^2\theta\cos^2\delta - \sin^2\theta\cos^2\theta\sin^2\delta - ie^{i\eta}\sin^3\theta\cos\theta\sin\delta\cos\delta)\left|0\right\rangle\left\langle 2\right|$$
$$+ (-ie^{i\eta}\sin\theta\cos^3\theta\sin\delta\cos\delta + \sin^2\theta\cos^2\theta\sin^2\delta - \sin^2\theta\cos^2\theta\cos^2\delta - ie^{-i\eta}\sin^3\theta\cos\theta\sin\delta\cos\delta)\left|0\right\rangle\left\langle -2\right|$$
$$+ (\sin^2\theta\cos^2\theta\sin^2\delta + ie^{i\eta}\sin^3\theta\cos\theta\sin\delta\cos\delta - ie^{-i\eta}\sin^3\theta\cos\theta\sin\delta\cos\delta + \sin^4\theta\cos^2\delta)\left|0\right\rangle\left\langle 0\right|$$
$$+ (ie^{i\eta}\sin\theta\cos^3\theta\sin\delta\cos\delta - \sin^2\theta\cos^2\theta\sin^2\delta + \sin^2\theta\cos^2\theta\cos^2\delta + ie^{-i\eta}\sin^3\theta\cos\theta\sin\delta\cos\delta)\left|2\right\rangle\left\langle 0\right|$$
$$+ (\cos^4\theta\sin^2\delta + ie^{i\eta}\sin\theta\cos^3\theta\sin\delta\cos\delta - ie^{-i\eta}\sin\theta\cos^3\theta\sin\delta\cos\delta + \sin^2\theta\cos^2\theta\cos^2\delta)\left|2\right\rangle\left\langle 2\right|\Big]$$

Only diagonal terms in a given basis will contribute to the randomness and by simplifying the above expression we quantify the randomness corresponding position space as,

$$R_i(\rho_2^p) = -\Bigg[(\cos^4\theta\cos^2\delta + ie^{-i\eta}\sin\theta\cos^3\theta\sin\delta\cos\delta - ie^{i\eta}\sin\theta\cos^3\theta\sin\delta\cos\delta + \sin^2\theta\cos^2\theta\sin^2\delta)$$
$$\times\ln(\cos^4\theta\cos^2\delta + ie^{-i\eta}\sin\theta\cos^3\theta\sin\delta\cos\delta - ie^{i\eta}\sin\theta\cos^3\theta\sin\delta\cos\delta + \sin^2\theta\cos^2\theta\sin^2\delta)$$
$$+ \sin^2\theta\ln(\sin^2\theta) + (\cos^4\theta\sin^2\delta + ie^{i\eta}\sin\theta\cos^3\theta\sin\delta\cos\delta - ie^{-i\eta}\sin\theta\cos^3\theta\sin\delta\cos\delta + \sin^2\theta\cos^2\theta\cos^2\delta)$$
$$\times\ln(\cos^4\theta\sin^2\delta + ie^{i\eta}\sin\theta\cos^3\theta\sin\delta\cos\delta - ie^{-i\eta}\sin\theta\cos^3\theta\sin\delta\cos\delta + \sin^2\theta\cos^2\theta\cos^2\delta)\Bigg]$$

Here an extra term is coming compared to coin space randomness expression because after 2-nd step in position space particle has three degrees of freedom in SQW scenario but for coin space it's being two. From here we can easily witness the advantage of using position space and benefit of using it with more number of steps.
